# Chitosan Nanoparticle-Based System: A New Insight into the Promising Controlled Release System for Lung Cancer Treatment

**DOI:** 10.3390/molecules27020473

**Published:** 2022-01-12

**Authors:** Cha Yee Kuen, Mas Jaffri Masarudin

**Affiliations:** 1 Department of Cell and Molecular Biology, Faculty of Biotechnology and Biomolecular Sciences, Universiti Putra Malaysia, Serdang 43400, Selangor, Malaysia; yeekuen_91@hotmail.com; 2 UPM-MAKNA Cancer Research Laboratory, Institute of Biosciences, Universiti Putra Malaysia, Serdang 43400, Selangor, Malaysia

**Keywords:** nanomedicine, chitosan nanoparticle, drug delivery systems, controlled release, lung cancer

## Abstract

Lung cancer has been recognized as one of the most often diagnosed and perhaps most lethal cancer diseases worldwide. Conventional chemotherapy for lung cancer-related diseases has bumped into various limitations and challenges, including non-targeted drug delivery, short drug retention period, low therapeutic efficacy, and multidrug resistance (MDR). Chitosan (CS), a natural polymer derived from deacetylation of chitin, and comprised of arbitrarily distributed β-(1-4)-linked d-glucosamine (deacetylated unit) and *N*-acetyl-d-glucosamine (acetylated unit) that exhibits magnificent characteristics, including being mucoadhesive, biodegradable, and biocompatible, has emerged as an essential element for the development of a nano-particulate delivery vehicle. Additionally, the flexibility of CS structure due to the free protonable amino groups in the CS backbone has made it easy for the modification and functionalization of CS to be developed into a nanoparticle system with high adaptability in lung cancer treatment. In this review, the current state of chitosan nanoparticle (CNP) systems, including the advantages, challenges, and opportunities, will be discussed, followed by drug release mechanisms and mathematical kinetic models. Subsequently, various modification routes of CNP for improved and enhanced therapeutic efficacy, as well as other restrictions of conventional drug administration for lung cancer treatment, are covered.

## 1. Introduction

Over the last decade, the incidence and mortality rate of cancer has remained one of the leading causes of death worldwide, second to cardiovascular diseases [1]. Based on the analysis of the World Health Organization (WHO), every sixth death in the world is due to cancer [2]. Approximately 1.8 million new lung cancer cases are discovered annually, and about 1.6 million deaths were reported among them, with 4%–17% 5-year survival rates liable on stage and regional difference [3,4,5]. This incidence rate is highly related to the uptake and consumption of tobacco according to gender and habits of people in different geographical areas [6]. Lung cancer can be classified into two main categories, small cell lung carcinoma (SCLC) that accounts for about 15% of all lung cancer, and non-small cell lung carcinoma (NSCLC), accounting for the other 85%. SCLC is known as an aggressive lung tumor that is heavily related to cigarette smoking, with patients often diagnosed with metastatic disease [7]. NSCLC is further sub-divided into adenocarcinoma (most common cases), squamous cell carcinoma, and large cell carcinoma [8]. Adenocarcinoma is most common in NSCLC, which arises from small airway epithelial cells, type II alveolar cells, which secrete mucus and other substances, while squamous-cell carcinoma arises from squamous cells in the airway epithelial cells in the bronchial tubes in the center of the lungs, and large cell carcinoma often arises in the central part of the lung [9]. Researchers have formulated several means of therapy such as surgery, radiation, and chemotherapy to treat cancer patients, but chemotherapy remains the most significant treatment among these approaches, with synthetic drugs such as anthracyclines being the most potent chemotherapeutic drugs used [10]. The four most prominent anthracyclines are epirubicin, doxorubicin, idarubicin, and daunorubicin [11]. Yet, the severe side effects following the chemotherapy due to non-specific destruction have become the main constrain for the treatment, including cardiotoxicity, nausea, and alopecia [12,13].

Arising from these issues, research focus has shifted to the utilization of nanotechnology to increase treatment efficacy and increase treatment safety. Polymeric nanoparticles are a large family which can generally be divided into natural and synthetic forms. The term polymer can be defined as macromolecules that are made up of bulky repeating units arranged in a chain-like structure, presenting an assortment of alignments, structures, and characteristics [14,15]. Natural polymers are comprised of two major types, polysaccharides- and protein-based polymers, whereas synthetic polymers include polyethers, polyesters, poloxamers, and recombinant protein-based polymer [14,16]. Although synthetic polymers have greater flexibility in terms of structure rationalization, natural polymers such as hyaluronic acid, chitosan, and alginate are still more desirable due to their biodegradability and biocompatibility, they are inexpensive and able to be modified [17,18,19]. Consequently, the utilization of chitosan nanoparticle (CNP) systems has been widely recognized by researchers due to their various advantageous characteristics, including being biocompatible, having low toxicity, are easy to prepare, and have tunable physical properties [20,21]. CS is one of the most prominent natural polyaminosaccharides acquired through the *N*-deacetylation of chitin, the second most abundant natural occurring polysaccharide after cellulose [22,23,24]. The structure of CS is composed of β-(1-4)-linked d-glucosamine and *N*-acetyl-d-glucosamine randomly disseminated within the polymer, enabling its cationic nature [25,26,27,28]. Besides, CS also has several features such as good biocompatibility and biodegradability, non-toxicity, and low allergenicity, which have fascinated researchers, and allow them to be utilized for numerous applications. Moreover, it was reported that CS possessed several biological properties such as anti-oxidant, anti-microbial, and anti-tumor activities [29,30]. However, there are numerous challenges of using these nanoparticles in anticancer treatments, such as their biocompatibility and toxicity in biological systems, the high attrition of anti-cancer agents in clinical trials, the slow progress of nanomedicines therapeutics to market, technical and cost involved in up-scaling and manufacturing, and poor understanding about the heterogeneity of disease in the patient population [31,32].

The CNP-based nanoparticles in drug delivery have been utilized in various diseases treatment, including cancer-related diseases, gastrointestinal diseases, pulmonary diseases, ocular infections, and drug delivery across the blood-brain barrier [33,34,35,36,37]. CNP possessed several features that enabled its utilization as a vital drug delivery vector, such as enhance delivery and therapeutic efficacy of drugs [38,39], improved intracellular accumulation [40,41], and controlled release properties [42]. Similar to other nanoparticulate delivery systems, CNP has a protective layer after the encapsulation of therapeutics into the core of nano-particles. The conventional methods of drug consumption such as oral delivery, sublingual delivery, and rectal delivery are always preferred by patients due to the ease of consumption widespread acceptance by patients [43]. However, the therapeutic efficacy of these medications is often less desired as the drugs that are consumed, especially orally, need to go through many different paths such as the digestive tract, gastrointestinal tract, and liver to reach the target site, which may lead to the cleavage or degradation by the enzymes or extreme pH environment before they reach the site of absorption and bloodstream [44,45]. Besides that, many drugs may become insoluble at low pH levels in the digestive tract, which may significantly reduce the bioavailability of the drugs to be absorbed into the bloodstream [46]. In order to overcome the obstacles faced by conventional methods, nano-mediated drug delivery systems such as CNPs have received more attention as a delivery system for drugs. The mucoadhesive properties of CNPs also contribute to the absorption and enhanced intracellular accumulation of drugs delivered by CNPs. As mentioned by Tiyaboonchai, both in vitro and in vivo experiments found that CNPs can enhance absorption through the combination of mucoadhesion and transient opening of tight junctions of the mucosal membrane [47]. The interaction between cationic CS and anionic mucin has been attributed to the mucoadhesive properties of CS, which elongates the contact time between the encapsulated drugs and the absorptive surface, and subsequently prolongs the half-life of drug clearance, which in turn enhanced the absorption [48]. Besides that, reports in the literature also show that positively charged CNPs exhibit electrostatic interactions with negatively charged cell membranes, thus revealing a greater uptake through the endo-cytosis pathway [49,50].

The study of Hassan et al. also demonstrated that CNPs promote intracellular accumulation by observing fluorescently-labeled glutamic acids (GA) with and without encapsulation by CNP under a fluorescence microscope. The results showed that the fluorescence signal was detected only in cells that were treated with with fluorescein isothiocyanate (FITC) labeled-GA (FITC-GA) encapsulated CS nanoparticles and was found mainly accumulated in the cell cytoplasm, while no fluorescence signal was detected in cells treated with free FITC-GA [51]. Additionally, CNP as a prominent drug delivery system has been reported to successfully encapsulate various therapeutics from chemotherapeutic drugs, such as doxorubicin, paclitaxel, and carboplatin, to natural anti-cancer compounds such as curcumin and hydrocaffeic acid [52,53,54,55,56,57]. As reported by Zare et al., doxorubicin encapsulated CNP was able to achieve nearly 90% of intestinal permeation post oral administration to the rat model [53]. Besides, Xu et al. has conducted an experiment that described the CNP encapsulation of paclitaxel to study the anti-cancer efficacy against DU-145 prostate cancer cell lines indicated increased growth inhibition effects with an increased concentration of paclitaxel [54]. Khan et al. also described the curcumin encapsulated CNP had enhanced water solubility, bioavailability, and cytotoxic efficacy against various cancer cell lines as compared with free curcumin [58]. On the other hand, Nashaat et al. utilized CNPs for the oral delivery of albumin. An in vivo experiment found that administration of albumin-loaded CNPs had greater enzymatic stability, sustained release, and higher serum concentration as compared with the free albumin [59]. The examples mentioned above have suggested CNP as a promising nano-carrier of anti-cancer therapeutics. Therefore, this review highlights multiple applications of CNP-based systems and their controlled release properties for delivering various therapeutic agents to attain high loading, enhanced therapeutic efficacy, pro-longed retention time, and overcome multidrug resistance (MDR) as effective lung cancer treatment modalities.

## 2. Current State of Chitosan Nanoparticles in Various Fields of Application

### 2.1. Advantages

**Figure 1:**
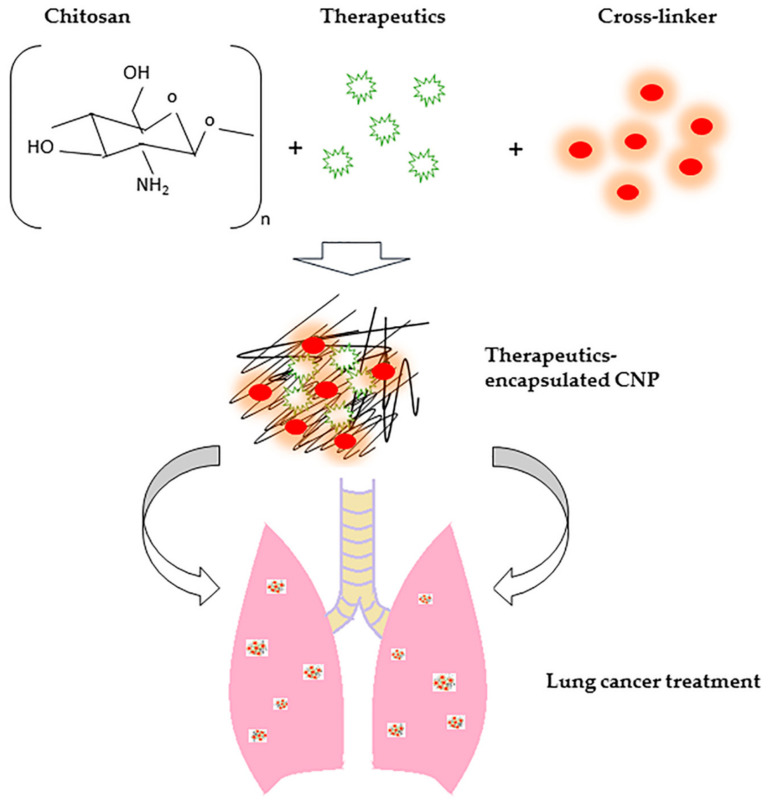
The illustration of the mechanism of chitosan nanoparticles (CNPs) for lung cancer treatment. The therapeutics were encapsulated into CNPs and delivered to the lung to achieve greater therapeutic efficacy.

The utilization of nanoparticles for the encapsulation of cargos such as various therapeutic drugs or compounds and genetic materials has been reported by many researchers over the years. As shown in Figure 1, for example, the brief mechanism of a therapeutic-encapsulated CNP system for lung cancer treatment has been illustrated. CS is one of the most prominently used natural polyaminosaccharides due to its characteristics, including good biocompatibility and biodegradability, ease of synthesis, non-toxicity, non-immunogenic, and applicable to a wide range of therapeutics [20,60,61,62]. Besides, as compared with other biodegradable polymers having a pharmacopeial monograph, CS is known as the only polymer that exhibits a cationic character, which resulted in its utilization as a drug delivery system [63]. Additionally, CS was known to possess inhibitory effects on proliferation of tumor cells, tumor-related angiogenesis, and metastasis, and thus demonstrating good anticancer activity [64]. Due to these advantages, it has fascinated the researchers to CNP in numerous applications. Previous toxicology evaluation study of CNP was conducted by Wang et al. on the embryonic development of zebrafish, and it was found that the zebrafish treated by CNPs with an average size of 84.86 nm had no significant mortality (<10%) at 120 h post-fertilization (hpf), even at the high concentration of 200 mg/L [65]. Additionally, this result was supported by another organ-specific toxicology study on zebrafish embryos by Abou-Saleh et al. They reported a 100% survival rate of embryos with no morphological and physiological abnormalities were observed after exposure to CNPs at 200 mg/L at 96 hpf [66]. Apart from that, CNPs have been reported to enhance the therapeutic efficacy of therapeutics, especially in tumor therapy, through passive targeting or enhanced permeation and retention (EPR) effects [67,68,69]. It has been suggested by the previous study of Ai et al. where copper (CuSO_4_) loaded CNPs showed greater therapeutic efficacy than free CuSO_4_ groups against osteosarcoma assessed through in vitro cytotoxicity assay, reactive oxygen species (ROS) analysis, and caspase 3 and 7 apoptotic activities analysis due to better internalization of CNP system [70]. On the other hand, On et al. also developed a non-toxic indocyanine green (ICG) and cyanine 5.5 (Cy5.5)-coupled tumor-targeting CNP-based system, which displayed outstanding tumor accumulation and prolonged biodistribution profiles in both rabbit squamous cancer cell (VX2) tumor-bearing mouse and rabbit models probably due to the EPR effects of CNP [71].

Moreover, it was reported that CS and CS derivatives possessed several biological properties such as anti-oxidant [72,73,74], anti-microbial [75,76,77], anti-inflammatory [78,79,80], and anti-tumor activities [81,82,83]. The anti-oxidation properties of CS were suggested to be closely related to its degree of deacetylation and molecular weight, where a lower molecular weight CS was more advantageous than higher molecular weight CS in the removal of free radicals while highly deacetylated CS had superior antioxidant activity compared to less deacetylated CS [84,85,86]. It was supported by previous studies that the free radical scavenging activity of CS may be attributed to the structure of CS with amino and hydroxyl groups attached to the C-2, C-3, and C-6 positions of the pyranose ring of CS, and higher radical elimination activity due to the presence of the high number of amino groups in highly deacetylated CS [87,88,89]. Additionally, the anti-microbial properties of CS have largely contributed to industry applications, especially in wound dressing, food preservation, and pollutants control [77,90]. In food preservation, CS has been widely studied to be used as edible food coating films to acts as an oxygen and low water vapor barrier for fruits and vegetables, extending their shelf life, and at the same time, reducing microorganism growth [91,92]. However, in the context of pollutant removal, Sun et al. proposed that CS had enhanced the antibacterial filter materials for ventilation applications against *Escherichia coli* showing its decent antimicrobial properties [93].

It has been further shown by a previous study of Al-Sherbini et al. that the development of CS/silver bionanocomposites has aided in photodegradation of organic pollutants, adsorption of heavy metals pollutants, and also showed antibacterial activities on both gram-negative (*E. coli*) and gram-positive bacteria ( gram positive. *bacillus*) [94].

### 2.2. Challenges

However, apart from the advantages of CS and its derivatives in various fields as mentioned above, there are numerous challenges remaining to be resolved to improve its use in the future. One of the main concerns is about the safety of CS and its derivatives, especially in biomedical applications. Generally, CS has been categorized as relatively safe due to its biodegradability and biocompatibility properties. It has been reported that elimination of CS was low molecular weight CS can be excreted through the kidney, while the high molecular form can be renally excreted after degraded into fragments [95]. Nevertheless, the utilization of the native form of CS is restricted, especially in wound management applications, mainly due to its water insolubility, high viscosity, and tendency to coagulate with proteins at high pH values [96]. Besides, Hu et al. has reported concentration-dependent toxicity of CNPs in zebrafish embryos [97]. It has also been suggested by the early study of Huang et al. that CNPs exhibited a cytotoxicity effect at concentrations higher than 0.741 mg/mL [98]. However, it was described by Sonin et al. that an extended 14 days of CNPs treatment in rats did not reveal any significant toxicity effects as assessed by hematological and biochemical parameters of the blood [99]. It has therefore inferred that the toxicity of CS might be due to the size of the CNPs and also the usage of different types of crosslinkers [100,101,102]. Glutaraldehyde, for example, is one of the highly effective cross-linking agents but is toxic to biological systems [103,104]. Besides, Parhi has also revealed that crosslinkers such as glutaraldehyde and glyoxal can potentially initiate toxicity in vivo [105].

### 2.3. Opportunities

Despite the challenges encountered by CS and its derivatives, the emergence of nanobiotechnology has opened opportunities in several fields, including agriculture, pharmaceutical, biomedicine, and the food industry [98]. Besides that, the CNP system has also contributed to several other fields of applications such as antimicrobial [106,107], pollutants removal [108], energy resources [109], and catalysis applications [110]. CNPs have been widely utilized in pharmaceutical and biomedicine fields due to their structural flexibility, leading to ease of modifications, conferred controlled release properties, provide protection for active components from degradation and localized retention [21,100,111]. In the pharmaceutical field, various drug delivery applications have been reported, including mucosal delivery, cancer drug delivery, anti-microbial drug delivery, ocular delivery, and vaccine delivery, which demonstrated the outstanding properties of CNPs to aid in the delivery of drugs/therapeutics [60,112].

Liu et al. reported a carboxymethylated CNP system to deliver the antiepileptic drug, carbamazepine, intra-nasally, which able to bypass the blood-brain barrier so as to enhance the brain drug concentration and therapeutic efficacy. This modified CNP system had nanoparticles 218.76 ± 2.41 nm in size with 80% high entrapment efficiency, and this system showed an increase in drug bioavailability and enhanced targeting properties through both in vitro and in vivo studies. [113]. Moreover, both the in vitro and in vivo investigation of Pawar and Jaganathan concluded that a glycol CNP system had high loading efficacy for hepatitis B vaccine and therefore suggested the potential of a CNP system for mucosal delivery of vaccines [114]. Additionally, evidence in the literature has shown that CNP systems have successfully aided in the delivery of cancer drugs [115,116]. Viravaidya-Pasuwat and colleagues developed an O-succinyl CS pluronic copolymer conjugated with an anti-HER2 monoclonal antibody for targeted delivery of doxorubicin (DOX) with enhanced therapeutic efficacy towards MCF-7 breast cancer cells [117].

Zare et al. have also shown that DOX encapsulated by a CNP system had about 12.7 fold greater intestinal permeation than that of free DOX, which suggested enhanced drug delivery by a CNP system [53]. On the other hand, paclitaxel (PTX) encapsulated in a CNP system has been reported and exhibited significantly greater anticancer properties than free PTX against MDA-MB-231 breast cancer cells. The PTX loaded CNP was 226.7 ± 0.70 nm in size and displayed sustained-release properties with enhanced cytotoxicity and apoptotic efficacy than the non-encapsulated counterpart [118]. A previous study by Aydin and Pulat proposed that 5-fluorouracil (5-FU) encapsulated CNP exhibited a pH-responsive controlled release pattern, while a recent study conducted by Smith et al. revealed that 5-FU encapsulated CNP possessed enhanced cellular uptake efficacy in HCT-116 colorectal carcinoma cells and significantly inhibited the growth of 2D and 3D HCT-116 spheroid model [119,120]. Besides, Chatzitaki et al. reported chitosan-coated polylactic acid-co-glycolic acid (PLGA) nanoparticles for the nasal delivery of ropinirole hydrochloride served as a potential therapeutic route for Parkinson’s disease treatment [121]. The recent study of Migone and colleagues also revealed a quaternary ammonium chitosan-methyl-β-cyclodextrin conjugate for delivery of neuropeptide dalargin (DAL). In vitro studies showed the fascinating ability of this nanoparticle system to deliver DAL across the blood-brain barrier to the central nervous system [122].

## 3. Drug Release Mechanisms of Nanoparticle Systems

The first introduction of controlled drug delivery was in 1952, which has progressed for more than six decades [123]. A controlled release drug delivery system is developed for the delivery and maintenance of drugs level within the minimum toxic concentration (MTC) and the minimum effective concentration (MEC) for an extended duration [124]. Controlled release properties have been comprised of extended-release, sustained-release, delayed-release, and targeted-release [125]. These advantageous properties of nanoparticles were assessed through two different measures, using drug release mechanisms and mathematical modeling models. These two different aspects are often correlated with each other and will be discussed in a later section.

### 3.1. Drug Release Mechanisms

Generally, the drug release mechanism of CNPs is divided into three different types. The first mechanism is the swelling of CS (swelling of polymer), diffusion of drug through polymeric matric or adsorbed drug, and degradation/erosion or combination of both degradation and erosion [113,126,127]. The swelling of a polymer, which results in pores creation, and also the diffusion of drugs from polymer surface have always been associated with the initial burst release occurrence in CNPs [128]. However, the CS derivatives have been suggested to effectively aid in changing the release rate of drugs from nanoparticles, providing tunable drug release, and influencing the pharmacokinetic profile of the encapsulated drug [129,130].

The diffusion-controlled drug release system was divided into monolithic- and reservoir-type, where both were generally initiated by drug permeated through the interior of the polymeric matrix and to the adjacent medium [131]. Interestingly, diffusion may be related to the swelling or erosion of a polymer. The diffusion-controlled release is also known as Fick’s law of diffusion, where described the solute transport route in which the relaxation time of polymer is much greater than the diffusion time of characteristic solvent [132]. The mathematical equation of diffusion is given by Fick’s law of diffusion, as shown in Equation (1) [132]

F = −D ∂c/∂x (1)

where F is the rate of transfer per unit area of section (flux), c is the concentration of the drug, and D is the diffusion coefficient (diffusivity). There are some assumptions to be made in order for the parameters of Fick’s law to be derived, which include the pseudo-steady condition is regulated throughout the drug release process, the mean distance of drug diffusion through the polymeric matrix is greater than the diameter of solute particles, and the maintenance of sink conditions are offered by the adjacent medium of the nanoparticles [133,134].

The swelling of the polymer is a volumetric-growth process in which denoted by the water imbibition into the polymer until the polymer dissolves [135,136]. The swelling of a polymer is dependent on various features, including the nature/amount of polymer, cross-linking density, the pH of the surrounding fluid, and temperature [135,137,138]. The polymer chains will experience disentanglement and subsequently initiated the release of the drug from the matrix when the polymer happenstance the adjacent medium and instigated polymer swelling. Additionally, the rate of drug absorption from the site of delivery in vivo is suggested to be correlated with the rate of drug availability for membrane transport or cellular uptake. It was elucidated by the previous study of Fonseca-Santos and Chorilli, that there are three important factors that contributed to the swelling-drug release profile, which are the hydrophilicity of the polymer, the swelling rate of polymer, and the density of the polymer chains [139].

The degradation and erosion release mechanism is amongst the most popular drug release mechanism as retrieval after the drug is released is not required due to the non-toxic and excretable characteristics of the delivery system [140]. Both degradation and erosion of polymers are correlated features, where degradation may often lead to succeeding physical erosion as bonds break. The erosion of polymer has been divided into two types, which are surface (heterogeneous) erosion and bulk (homogeneous) erosion since the early 1980s [141,142].

Bulk erosion occurs when water invades the polymer more rapidly than hydrolysis can occur [143]. Under these circumstances, the polymer chain scission processes will take place throughout the polymer due to the presence of water throughout the matrix. The rate of initial hydrolysis may be very slow due to the length of polymer chains, while the initial scissions may limit the hydrolysis since it will provide high mobility to the chains, which allows the migration of chains and formation of crystallites. Nonetheless, the hydrolysis may be augmented after a certain degree of chains have been hydrolyzed. Additionally, the erosion will be autocatalytic in case that the chain scission occasioned the formation of acidic end groups [144]. Herein, the drug release of polymers through bulk erosion can be summarized in three different phases. In the first phase, the drug is released from the exterior of the device or from pores that are linked to the surface. The second phase describes the initial degradation of the polymer, and the remaining drug is trapped in the device. In the last stage, the polymer is degraded, and the trapped drug is released rapidly from the device [132].

Meanwhile, the occurrence of surface erosion can be initiated by both conditions were either when water invasion is slow or when hydrolysis is rapid, which starts at the exposed surface and moves downwards [145]. Polyanhydride is one of the model polymers that undergo surface erosion, which has extraordinarily hydrophobic polymer characterized by repeating units of anhydride groups in the polymer backbone [146]. The hydrolytically labile anhydride bonds of polyanhydrides in the interior of the polymer matrix are evaded from water, and the drug release with hydrolysis can happen only at or adjacent to the surface [147]. Thus, it has indicated that the remarking characteristic of surface erosion is that device dimensions decrease with time. However, the rate of erosion may vary between different sizes and shapes of devices, which was correlated with exposed surface area [144,148].

### 3.2. Mathematical Modeling Fitting of Drug Release Kinetics

Generally, the controlled-release mechanism can be categorized into several forms, including diffusion, swelling, and erosion, as mentioned above. Consequently, various types of nanomaterial with different physical and chemical properties and also the nature of the encapsulated drugs will be released through different routes. Therefore, it is vital to identify the release mechanism of drugs/therapeutics and ensure the release in a controlled manner. To date, various mathematical models have been utilized for the fitting of different drug release mechanisms. Among these models, zero-order, first-order, the Higuchi model, the Korsmeyer-Peppas model, and the Hixson-Crowell model have often been used as the models for the drug release fitting of nanoparticles and will be further discussed in the following sections [149,150]

#### 3.2.1. Zero-Order

Theoretically, the drug delivery system that has a zero-order drug release profile is the ideal system and pursued by researchers to achieve a low dosing rate with a uniformly release profile without initial burst release throughout the entire release duration [151]. The respective mathematical equation is shown in Equation (2)

Q_t_ = Q_0_ + K_0_t, (2)

where Q_0_ = initial amount of drug; Q_t_ = cumulative amount of drug release at time “t”; K_0_ = zero-order release constant.

This elimination of zero-order kinetics takes place at a constant rate following a linear elimination phase as the system becomes saturated, independent of the plasma concentration [152]. However, in reality, there are several restrictions that limited drug release to achieve a zero-order profile, including the properties and matrices of the nanomaterials, the influence of the external environment, and the properties of the cargos [153]. Thus, there are various release models such as a first-order model, Higuchi model, Hixson-Crowell model, and Korsmeyer-Peppas model that have been modulated.

#### 3.2.2. First-Order

The first-order model was first suggested by Gibaldi and Feldman in 1967 and later by Wagner in 1969 [154]. The mathematical equation is shown in Equation (3)

log Q_t_ = log Q_0_ + K1t (3)

where Q_t_ is the cumulative percentage amount of drug released at time t; Q_0_ is the initial amount of the drug; K1 is the first-order release constant; t is time.

It has been commonly employed to define the absorption and/or elimination of an assortment of therapeutic agents. The elimination in first-order kinetics dependent on the concentration of only one reactant (drug) and the drug is eliminated at a constant fraction per unit of time, which also means that the elimination will increase proportionally as the plasma concentration increases, following an exponential elimination phase as the system never attains saturation [150]. Nonetheless, it is a simple model and widely adapted to various nano-carriers. The previous study of Smits et al., for instance, has revealed that the release of liposomal prednisolone phosphate in mice following in vitro administration in mice followed the first-order release profile for all experimental tissues study [155].

#### 3.2.3. Higuchi Model

Next, the Higuchi model was described by Higuchi in 1961 on his study of the rate of release of ointment bases containing drugs in suspension [156]. The mathematical equation of the Higuchi model is presented in Equation (4)

Q_t_ = Q_0_ + K_H_t^1/2^ (4)

where Qt is the cumulative percentage amount of drug released at time t; Q_0_ is the initial amount of the drug; K_H_ is the Higuchi constant; t is time.

This model appeared as one of the most famous and most frequently utilized models for the release of cargos from matrix systems. There are several important rules that must be followed in this model: (I) the initial concentration of therapeutic existed in the matrix is much greater than its solubility; (II) the diffusion of therapeutic happens only in one direction where the edge effect is negligible; (III) the thickness of the system is much greater than the size of the therapeutic; (IV) the swelling and/or dissolution of the matrix is insignificant; (V) the diffusivity of therapeutic is persistent; (VI) the perfect sink circumstances are achieved [157].

#### 3.2.4. Hixson-Crowell Model

On the other hand, the Hixson-Crowell model is another well-known release model that was revealed by Hixson and Crowell in 1931 upon the discovery of a collection of particles’ fixed area is comparative to the cube root of its volume, while its mathematical equation is presented in Equation (5) [150]

∛Q_t_ − ∛Q_0_ = K_HC_t (5)

where Q_t_ is the cumulative percentage amount of drug released at time t; Q_0_ is the initial amount of drug at time t; K_HC_ is the Hixson-Crowell constant.

This model is commonly used for pharmaceutical systems such as tablets, in which the dissolution rate is equivalent to the surface of the dosage form; the surface area erodes consistently over time while the geometrical form remained unchanged. A previous study conducted by Malana and Zohra showed the in vitro release study of tramadol hydrochloric acid from chemically cross-linked ternary-polymeric hydrogels matrix tablet [158]. The study revealed that formulations of different concentrations of polymers and drug payloads were well suited to the Hixson-Crowell release model.

#### 3.2.5. Korsmeyer-Peppas Model

Additionally, the Korsmeyer–Peppas model was derived from the Power law, which is a more comprehensive model to term the drug release from a polymeric system. This model was established by Korsmeyer et al. in 1983 to describe the exponential relationship between the release of drug and the time where its mathematical equation was shown in Equation (6) [159]

Qt = K_KP_t^n^, (6)

where Qt is the cumulative amount percentage of drug released at time t; K_KP_ is the Korsmeyer-Peppas constant; n is the release exponent describing the drug release mechanism.

This Power Law model is advantageous for the study of polymeric drug release systems, especially when the release mechanism is unfamiliar or when multiple release mechanisms are involved [160].

This section has summarized the various drug release mechanisms of nanoparticulate drug delivery systems. The drug release of polymeric nanoparticles was categorized in diffusion, swelling, and degradation/erosion, which are characterized differently by the composition, ratio of composition, interactions between the components, and preparation methods [161]. Apart from that, the rate of drug release has been fitted into various mathematical modeling equations as mentioned above, where the controlled release of drugs from nanoparticles over an extended period of time in a controlled drug release system [162]. Subsequently, numerous modifications of CS will be discussed in the next section to discuss its potential to be used as controlled release delivery vehicles.

## 4. Modification of Chitosan Nanoparticle Systems

Despite the advantages of CNP as a great nano-carrier system, there are some limitations that exist, such as low encapsulation efficiency for poorly water-soluble compounds and low solubility in physiological pH, which limits the usage of this system [163,164]. For example, CS itself has poor solubility in a solution above pH 6 and has poor encapsulation for hydrophobic drug candidates, which require glycol modifications to the CS to attain greater encapsulation and delivery efficiency [165]. In order to overcome these shortcomings, various modifications of the traditional CNP system have been carried out by researchers. The modifications were generally categorized into two classes which are physically and chemically routes to alter the features like stability, mucoadhesion, and solubility for different purposes [166].

### 4.1. Physical Modification of Chitosan Nanoparticles

The physical modification of CS nanoparticles involves physically mixing two or more polymers to create a new, improved material with altered physical characteristics, including chemical, structural, and biological properties [167,168]. Some common polymers utilized for physical modification include polyvinyl alcohol, polyethyl oxide, and polyvinyl pyrrolidone [169]. For example, Risbud et al. used poly(vinyl pyrrolidone) (PVP)-modified CS nanoparticles to develop a hydrogel to encapsulate amoxicillin, an effective antibiotic in treating peptic ulcers caused by *Helicobacter pylori* [170]. The antibiotic was effective under in vitro conditions but scored poorly in in vivo situations which was suggested to be due to sub-effective bactericidal concentrations available at the site and their instability after oral administration. However, the CS-PVP, semi-interpenetrating polymer network (semi-IPN)-based controlled release antibiotic delivery system was found to be well-suited for use in a gastric environment. Moreover, a previous study of the development of curcumin encapsulated CS-polyvinyl alcohol silver nanocomposite to achieve enhanced anti-microbial activities [171]. On the other hand, Smith et al. have proposed a rapid self-assembled and physically cross-linked poly(ethylene glycol) (PEG)ylated CNPs in a single step manner by using the Flash NanoPrecipitation route to develop a high monodispersity nanoparticle system [172]. On the other hand, a recent study conducted by Liu et al. also demonstrated a PVP-modified amphiphilic CNP system for delivery of paclitaxel with enhanced antitumor inhibition in in vivo mice models without significant sub-acute toxicity [173].

### 4.2. Chemical Modification of Chitosan Nanoparticles

**Figure 2:**
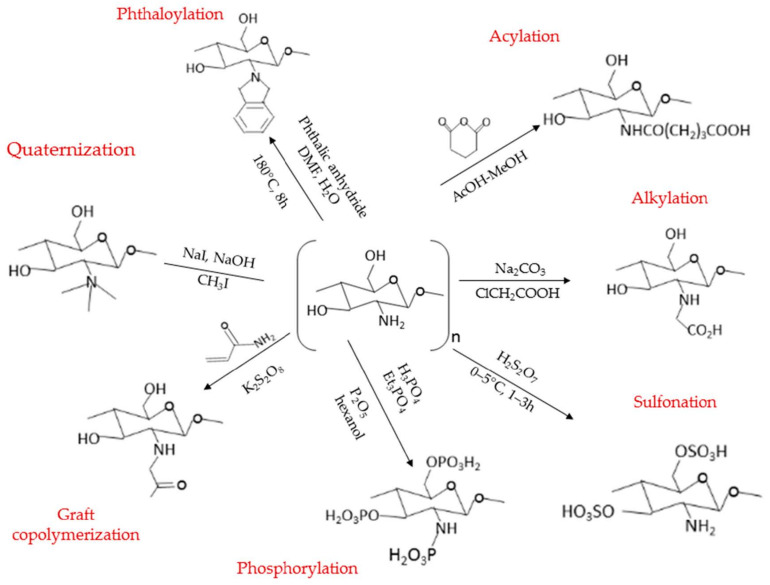
The various modification routes of chitosan (CS). Figure and reaction conditions were adapted from the study of Sajid et al., with modifications [174].

Subsequently, the chemical modification of CS can be achieved by modifying the primary amine groups of CS through chemical, photochemical, radiation, enzymatic grafting, and plasma-induced methods. Due to the presence of the large amount of amino (-NH_2_) and hydroxyl (-OH) groups with chemical activities in CS, numerous well-known CS derivatives have been developed by researchers through acylation, alkylation, quaternization, phosphorylation, graft copolymerization, phthaloylation, sulfonation, carboxymethylation, and carboxyalkylation of CS, as shown in Figure 2 [174]. Generally, the most common chemical modification route for CS is through *N*-substitution, where the reaction occurs through the amino functional group of CS [175]. Additionally, the *O*-substitution of CS, where the reaction occurs through the hydroxyl functional group, is also a common route for chemical modification of CS [176,177]. Yet, the *O*-substitution of CS often requires protection and deprotection of primary amino groups due to the higher reactivity of amino groups than hydroxyl groups [178,179]. The *N*,*O*-substitution that is involved in the substitution of both the amino and hydroxyl functional groups of CS will also usually provide an amphipathic property, and at the same time, can be used to enhance the hydrophobic and hydrophilic features of CS [180].

A previous study demonstrated that quaternized *N*-trimethyl CS chloride is a great candidate for enhanced transport of hydrophilic compounds across intestinal Caco-2 cell monolayers and proposed it to be an intestinal absorption enhancer for hydrophilic macromolecules [181]. Besides, Zhu et al. have also conducted thiolation modifications to CNPs to prepare thiolated CS sodium alginate nanoparticles and accomplished a greater mucoadhesive feature, higher stability, and a more effective treatment than the unmodified version towards human corneal epithelium cell lines [182]. On the other hand, the modifications to the CNP system have been reported to be capable of improving its drug delivery properties. As reported by Elgadir et al., various modifications of CNP can subsequently enhance the drug delivery properties comprising gene expression properties, mucoadhesive properties, and permeation enhancing properties [112]. It was supported by the study of Jintapattanakit et al. that PEGylated trimethylated CNPs enhanced their mucoadhesive properties up to 3.4-fold, which enhances its interpenetration and, in turn, its delivery properties [183]. It was further supported by evidence that thiolated CNP has shown penetration enhancement properties than unmodified CNP, which, in turn, indicated an absorption enhancing effect of a nano-carrier system in intestinal tissue [184].

The discussion in this section has shown various examples of modification, which suggests that CNP-based systems can be further modified for improvement to attain functionalization purposes, including solubility, higher encapsulation efficiency, and therapeutic efficacy. As shown in Figure 2, several examples of modification can be pursued for CS, including methylation, *N*-alkylation, and *N*-acylation of CS. These modifications of CS have exerted additional features to the CNP system for enhanced delivery and efficacy of drugs. Besides that, Table 1 has also suggested that CNP holds great potential as a prominent system to be utilized and applied to various kinds of drugs and in a wide range of sectors. Henceforth, evidence from the literature reveals that CS and its derivatives have high elasticity and potential due to their flexibility and efficacy in improving the current state of pharmaceutical and biomedical applications. Additionally, the controlled release properties of CNP or modified CNP system can also further enhance the efficacy of various drugs and reduce the concomitant effects of traditional drug administration routes, and this will be further discussed in the following section.

## 5. The Role of Controlled Release Chitosan Nanoparticle Systems for Lung Cancer Treatment

To date, innumerable modalities such as therapeutic drugs, peptides, DNA, siRNA, and vaccines have been delivered using CNP-based nanoparticle systems [112,197,198,199]. Additionally, CS and its derivatives have been suggested applicable in the recent COVID-19 pandemic infection treatment, characterized by acute respiratory distress syndrome due to their molecular weight, degree of substitution, and substituent type [200,201]. The encapsulation of therapeutics by nanoparticles can potentially increase the bioavailability and cellular uptake and can thereby reduce the total dosage of therapeutics to be consumed by patients [202]. Subsequently, the applications of a CNP system in lung cancer therapy and its controlled release properties benefited from the increased loading, increased duration of treatment, and reduced multidrug resistance will be discussed in the following section.

### 5.1. Increased Loading and Therapeutic Efficacy

The CS is soluble in a dilute acidic environment, and the basic amino groups will be protonated to the ionizable soluble R-NH_3_^+^ form with a positive charge [203,204]. The mucoadhesive properties of CS, due to its cationic charge nature, give it enhanced adherence to the mucosa of lung epithelial cells and extended the release of encapsulated therapeutics [205,206,207]. Our research group has previously studied the encapsulation of two different natural compounds, namely silibinin and protocatechuic acid, using a palmitic acid hydrophobically modified-CNP system. In vitro studies have found that the therapeutic efficacy of these two low solubility natural compounds was enhanced against the A549 human lung cancer cell line with about a one-fold increment of drug loading with sustained-release properties compared to their unmodified counterparts. [83,163]. These outcomes have suggested that the drug dosage used for disease treatment can be reduced to prevent the undesirable concomitant effects bring along with high drug concentrations. The recent study of Othman et al. was found to be congruent with our findings, where single- and dual-loading of L-ascorbic acid and thymoquinone into the palmitic acid-CNP system were enhanced and presented controlled release properties [208]. On the other hand, the previous study of Almutairi et al. showed that the encapsulation of raloxifene, an FDA-approved anti-cancer drug with hyaluronic acid-decorated CNPs, could effectively induce cytotoxic effects and apoptotic death in the A549 cell line as compared with the free drug at the same concentration [209]. Additionally, Babu et al. has shown an effective RGD (arginine-glycine-aspartic acid) peptide-modified polylactic acid-co-glycolic acid (PLGA)-CNP system for delivery of integrin αvβ3 receptor-targeted PTX towards non-small cell lung cancer (NSCLC) cells as compared with free drug and non-targeted counterparts and possessed negligible effects on normal human bronchial epithelial (NHBE) cells [210]. This evidence from the literature, therefore, demonstrates the outstanding efficacy of CNP-based systems in increased drug loading and therapeutic efficacy for lung cancer treatment.

### 5.2. Increased in Duration/Time Persistence

The elongated therapeutic duration endowed by CNP has been suggested to be strongly associated with their controlled-release properties [211,212,213]. Various release kinetic model has been introduced in the previous section, while the advantages and some examples of controlled-release CNP-based systems in drug delivery will be discussed at this point. According to Bhowmik et al., the advantages of a controlled release system has included the reduced in the fluctuation of drug concentration in blood, reduced in total drug required as compared with the conventional route, which can, in turn, reduce the local and systemic drug toxicity, and the reduced of administration frequency which can lead to improved patient obedience [214]. Consequently, the utilization of the CNP-based system has emerged as one of the most popular drug delivery vehicles. This biocompatible system has not only been employed for drug delivery but also in several biomedical and pharmaceutical applications, including anti-microbial applications, tissue engineering, cancer treatment, imaging, and gene delivery [64,72,215,216]. One of the key factors that have led to the development of these applications is the controlled release properties of CNP-based systems that prolong the drug release duration after administration [217,218].

Wang et al. have previously developed a folic acid conjugated poly(ethylene glycol) CNP (FA-PEG-CNP) system for the delivery of gemcitabinein (GEM) for lung cancer treatment. The in vitro assessment revealed GEM sustained release properties of up to 10 days while their use in an in vivo tumor-bearing female Balb/c mouse model indicated the distribution of GEM delivered by FA-PEG-CNP system was significantly higher in A549 tumor compared to other organs further outlined the longer residence of GEM in the target organ [219]. Besides, Rosiere et al. have described the synthesis of folate-grafted CS solid lipid nanoparticle system to enhance the delivery of paclitaxel for lung tumor therapy via inhalation route and able to penetrate the M109-HiFR murine lung carcinoma cell subline in vivo [220]. Fascinatingly, this study has revealed a prolonged pulmonary exposure of up to 6 h to paclitaxel with the limited systemic distribution. Additionally, Chen et al. have highlighted the prolonged drug release of methotrexate (MTX) and pemetrexed (PMX) dual drug-loaded methoxy poly(ethylene glycol) modified-CNP (MTX-PMX-pCNPs). The in vitro release study has revealed extended sustained release behavior of drugs for up to six days, and the cell viability assessment has shown that MTX-PMX-pCNPs have a significantly higher cytotoxicity effect towards the A549 cell line, especially in the prolonged incubation time points as compared with the non-encapsulated counterpart [221]. Thus, these previous studies have presented the efficacy of the CNP-based system for the prolonged release of therapeutics and protruded its potential to be employed as delivery vehicles for lung cancer treatment.

### 5.3. Mitigation of Multidrug Resistance (MDR)

Multidrug resistance (MDR) was first discovered in bacterial strains upon the discovery of penicillin in 1928 and was followed by a huge number of antibiotics [222,223]. One of the most critical concerns that arose from this discovery was methicillin-resistant *Staphylococcus aureus* (MRSA), which is resistant not only to methicillin but also to many other classes of antibiotics and disinfectants, and became the main source of hospital-acquired infections [224,225]. A similar situation occurs in cancer therapy, where conventional chemotherapeutics often encounter the problem of MDR and account for more than 90% of the mortality in cancer patients [226,227,228]. This state of resilience against mechanistically and structurally distinct drugs that may be intrinsic (primary) or acquired (secondary) during treatment, as a response to chemotherapy [229,230,231]. Statistical analysis has shown that most of the chemotherapeutics have significant initial therapeutic efficacy, but the majority of patients soon develop resistance at the latter stage of treatment. For instance, approximately 30%—55% of NSCLC patients develop recurrence and subsequently die from the disease [232,233]. Besides, nearly 20% of pediatric acute lymphoblastic leukemia patients relapse [234,235]. About 50%—70% of ovarian adenocarcinomas also reoccur in 1 year after treatment [236,237]. one of the major routes strategized to modulate MDR was the increased intracellular concentrations of drugs in MDR cells, and may possibly elicit severe subsequent concomitant effects in patients [238,239]. Consequently, CNP-based system has been studied in order to overcome the impediment of MDR.

The previous study of Zhang et al. has established an α-tocopherol succinate-modified CNP system to encapsulate paclitaxel (PTX)-d-α-tocopherol succinate prodrug for the enhanced loading and delivery of paclitaxel and against the MDR of tumor cells. This CNP-based system has been shown to be effective at initiating the reversal of MDR through decreasing mitochondrial membrane potential (MMP), inhibiting ATP synthesis, and suppressing P-glycoprotein (P-gp) expression, which are characteristic mechanisms of MDR [240]. Apart from that, Huang and coworkers have shown that dual drug-loading of cisplatin and demethoxycurcumin into a CD133 antibody surface-modified CNP system was able to achieve high efficacy synergistic effects against A549-ON cell line, which presenting stem-like characteristics and overexpression of CD133, and prepared via transfection of A549 cells by a lentiviral infection system with vectors encoding Oct4 and Nanog cDNA [241,242]. Since the cancer stem-like cells were known for their MDR features and their roles in metastasis and cancer reoccur after treatment, the outcome of Huang et al. has suggested an effective approach to combat MDR lung cancer cells [243,244]. Moreover, Nascimento et al. have formulated a siRNA encapsulated epidermal growth factor receptor (EGFR)-targeted CNP system through PEG conjugation [245]. The in vitro studies have revealed a decent effect in the silencing of the *mad2* gene in the A549 cell line, which is an essential gene that is responsible for the precise chromosome segregation during mitosis and has served as an alternative in MDR lung cancer treatment. In this context, Table 2 has listed several studies of CNP-based nanoparticles as drug delivery system, the drug-loaded onto the system, and their respective outcomes. It has presented the enhanced drug loading and/or therapeutic efficacy by the CNP-based system, especially in lung cancer treatment, which shown the relevance with the previous section.

## 6. Conclusions

Present cancer therapeutic approaches have encountered various circumstances that often resulted in limited therapeutic efficacy. The underlying basis of the failure is multifactorial, comprising short retention time of drugs, non-targeted delivery of drugs, and MDR. CNPs have been proven to be a promising nano-carrier system by previous studies due to their advantageous features in different sectors, especially in the biomedical field, in which they are capable of enhancing the therapeutic efficacy and cellular uptake of several anti-cancer drugs. Noticeably, the development of a CNP-based system has relieved the disadvantages of conventional cancer disease treatment with enhanced therapeutic efficacy and reduced MDR. The CS structure endowed with high flexibility has made it possible for it to be modified and given increased functionalization, overcoming the impediments in current pharmaceutical and biomedical applications. Nonetheless, further ongoing research on CS-related nanobiotechnology should be conducted in order to improve the current medical level and to develop medications with high therapeutic efficacy so as minimize the pain that patients have to endure and at the same time maximize the efficacy of therapeutic drugs.

## Figures and Tables

**Table 1:** Applications of chemically-modified CNP-based systems.

CS Derivatives	Application	Reference
Methylated *N*-Aryl CS Derivatives	An enhanced agent for in vitro paracellular permeation and in vivo adjuvant activity post oral administration to mice.	Suksamran et al. [185]
Quaternary CS magnetic composite modified with ammonium salt and combined with iron (II and III) oxide (Fe_3_O_4_) nanoparticles	A pH-dependent bioadsorption agent for methyl orange and chromium (VI) with homogeneous monolayer chemisorption behavior process.	Li et al. [186]
Acylated *N,N,N*-trimethyl CS nanopolyplexes associated with single-stranded oligonucleotides	Stable and nontoxic nanopolyplexes with enhanced cell transfection efficiency towards HeLa/Luc705 cell line.	Santos et al. [187]
*N*-acylated CS with glutaric anhydride in an aqueous acetic acid-methanol	Water soluble *N*-(4-carboxybutyroyl) CS derivative with improved antibacterial activity against *Agrobacterium tumefaciens* and *Erwinia carotovora*. and antifungal activity against *Botrytis cinerea*, *Pythium debaryanum*, and *Rhizoctonia solani*	Badawy and Rabea. [188]
*O*-(3,6-hydroxyethyl) CS	Improved water solubility, anticoagulation activity, and antibacterial activity against *Escherichia coli*.	Liu et al. [189]
Poly(ethylene glycol)-grafted-CS hydrogel	An improved delivery vector for delivery of T lymphocytes for brain tumor immunotherapy and potentially improved the glioblastoma immunotherapy.	Tsao et al. [190]
Polyurethane-grafted-CS copolymer	A biocompatible and hemocompatible copolymer with sustained release of tetracycline hydrochloride fitted with the Korsmeyer-Peppas release model.	Mahanta et al. [191]
3,6-*O*-sulfonated CS	Human papillomavirus (HPV) infection inhibition via directly HPV capsids binding or indirectly blockage by host PI3K/Akt/mTOR pathway interference to prevent the entry of HPV16 through cell autophagy.	Gao et al. [192]
2-hydroxyethylacrylate-grafted-CS	Controlled release of levofloxacin and proposed promising solution for topical wound management.	Siafaka et al. [193]
CS-*N*-2-hydroxypropyl trimethyl ammonium chloride	Enhanced water solubility and moisture-retention capacity, with antimicrobial activities against, *Staphylococcus aureus*, *S. epidermidis*, *Bacillus subtilis*, and *Candida albicans.*	Chi et al. [194]
Amino acid-grafted and *N*-acylated CS thiomers three-dimensional hydrogel scaffolds	A promising cytocompatible three-dimensional bio-scaffolds for potential cartilage repair applications.	Borsagli et al. [195]
Glutaraldehyde cross-linked carboxymethyl CS	Biocompatible with good hemostatic effect with an improved healing effect on liver injury in rats.	Zhang et al. [196]

**Table 2:** The outcome of drug-loaded CNP-based system for enhanced therapeutic efficacy/prolonged retention time/anti-MDR.

CNP-Based System	Drug Loaded	Outcomes	Reference
Native CNP system	Curcumin (Cu)	In vitro studies revealed enhanced permeation of Cu through Strat-M^®^ membrane and possessed controlled release properties in both pH 5.0 (first-order) and pH 7.4 (Higuchi) with negligible cytotoxicity towards human keratinocyte (HaCat) cell line compared to free Cu.	Nair et al. [56]
Native CNP system	Endostatin (ES)	The encapsulation of ES into CNP has prolonged the retention time of ES in vivo. The combined treatment of Lewis lung carcinoma (LLC) mouse Xenografts using ES-CNP and paclitaxel had significantly improved antitumor efficacy through suppressed proliferation and angiogenesis in the tumor tissues.	Xie et al. [246]
Native CNP system	Alphastatin (As)	In vitro studies have revealed high stability of the system and the sustained release properties of As for up to six days. Next, in vivo study of subcutaneous LA975 lung carcinoma xenograft in a T739 mouse model showed the greatest antiangiogenic effects and good hemocompatibility compared to the non-encapsulated counterparts.	Zhang and Hu [247]
Native CNP system	Docetaxel (Doc)	The in vitro studies showed the release of Doc from Doc-CNP using sustained-release proparticles (Higuchi release kinetic model) and possessed a significantly higher cytotoxicity effect in both dose- and time-dependent manners towards A549 cells compared to free Doc. The in vivo study using A549 xenograft nude mice also shown an enhanced anti-proliferative effect as compared with free Doc.	Nair and Velmurugan [248]
Folic acid conjugated CNP system (FLA-CNP)	Temozolomide (TMZ)	The TMZ-FLA-CNP was found to possess controlled- and sustained-release of TMZ with the highest antiproliferative efficacy against A549 lung cancer cell line as compared with TMZ-CNP and free TMZ. In vivo studies using A549 xenografted BALB/c-nu/nu athymic mice showed targeted delivery by pulmonary deposition and significantly higher tumor growth suppression as compared with TMZ-CNP and free TMZ.	Li et al. [249]
CS/ poly(lactic acid)/ graphene oxide/TiO_2_ composite nanofibrous scaffolds (CS/PLA/GO/TiO_2_)	Doxorubicin (Dox)	The CS/PLA/GO/TiO_2_/Dox showed the controlled-release of Dox (Korsmeyer–Peppas release kinetic model) for up to 14 days. The anti-proliferation efficacy of Dox was enhanced by the scaffolds and augmented with higher CS/PLA/TiO2/DOX/GO nanofibers concentration and the presence of magnetic field towards the A549 cell line.	Samadi et al. [250]
Carboxymethyl dextran conjugated CNP (CMD-CNP)	insulin-like growth factor 1 receptor specific siRNA (IGF-1R-siRNA) and doxorubicin (Dox)	The in vitro studies have presented the dual drug loading of IGF-1R-siRNA and Dox by CMD-CNP has enhanced the anti-migration, cytotoxicity, and apoptosis efficacy against A549 cell line as compared with a single-drug loading and free drug counterpart.	Shali et al. [251]
CS/poly(ethylene glycol)-anisamide (CTS/ PEG-AA) system	Gemcitabine (GEM)	The CTS/PEG-AA system showed the sustained-release of GEM for up to 15 days with enhanced in vitro cellular uptake and the most competitive cytotoxicity efficacy against A549 cells compared to CTS/PEG and free GEM. The in vivo study also showed superior tumor suppression against A549 subcutaneous tumors in mice in which revealed targeted delivery of GEM to the target site.	Garg et al. [252]
Native CNP system	Curcumin (Cu)	The Cu-CNP was released in a sustained-release manner for more than seven days and found with greater cytotoxicity efficacy even compared with Cu dissolved in DMSO solvent against HT1299 human lung cancer cell line. The in vivo studies using Swiss albino mice revealed that Cu-CNP possessed enhanced lung localization and were more competent in preventing benzo(a)pyrene-induced lung cancer as indicated by downregulation of proliferating cell nuclear antigen (PCNA), expression of p65 expression pERK.	Vijayakurup [253]
Nucleolin-targeting aptamer AS1411 and luminescent gold nanoclusters (AuNCs) functionalized CNP (AuNCs-CS-AS1411) system	Methotrexate (MTX)	MTX@AuNCs-CS-AS1411 showed the greatest in vitro anticancer effect against A549 cells compared to free MTX and MTX@AuNCs-CS as specified by an apoptotic death analysis. In vivo studies using A549 xenografted BALB/C nude mice have shown that AuNCs-CS-AS1411 conferred targeted delivery of MTX and effectively suppressed the tumor growth compared to free MTX.	Guo et al. [254]
Folate and carboxymethyl-β-cyclodextrin grafted trimethyl CNP system	Doxorubicin (Dox) and siRNA	Apart from showing satisfactory encapsulation efficiency, the grafted CNP system possessed pH-dependent controlled sustained release properties and significantly enhanced the therapeutic efficacy of the drugs,	Zhang et al. [255]
Cetuximab conjugated CNP (Cet-CNP) system	Quercetin (QUE) and Paclitacel (PTX)	The Que encapsulated Cet-CNP synergistically improved the cytotoxicity of PTX in A549 and reversed resistance in PTX resistant A549/Taxol cells. Besides, in vivo study revealed that the Que-PTX dual-loaded Cet-CNP had tumor growth inhibition in PTX-resistant xenografts.	Wang et al. [256]
Chitosan coated PLGA nanoparticles (CS-PLGA-NP) system	Resveratrol (RES)	The CS-PLGA-NP system showed improved stability and sustained release properties. Besides, the RES encapsulated CS-PLGA-NP showed significantly greater (about one-fold) cytotoxicity and apoptotic activities against H1299 lung cancer cells than the free drug counterpart.	Aldawsari et al. [257]
Native CNP system	*Morinda citrifolia* essential oil (MCEO)	The MCEOs-encapsulated CNPs had more than one-fold lower IC_50_ values as compared to free MCEOs (95 μg/mL, and 40 μg/mL, respectively) against A549 cells.	Rajivgandhi et al. [258]
Native CNP system	boswellic acid (BWA)	The BWA encapsulated CNP showed enhanced therapeutic effects against A549 cells because of greater cellular uptake, sustained-release properties, and enhanced antiproliferative effects as compared with free BWA.	Solanki et al. [259]
Hydrophobic deoxycholic acid (DCA) poly(amidoamine) dendronized CNP (DCA-PAMAM-CNP) system	Doxocubicin (Dox) and pDNA	The co-delivery of Dox and pDNA by using the DCA-PAMAM-CNP system achieved a high transfection efficiency of up to 74% in the 293T kidney cell line. Besides, a low dosage of co-delivered Dox was capable of improving transgene expression, presenting a synergistic effect.	Chen et al. [260]
Native CNP system	Anti-programmed cell death protein ligand 1 (aPD-L1)	The aerosol inhalation delivery system of aPD-L1 by CNP has been suggested to be a potent immunotherapy against lung metastasis through activation of the immune system by promoting the infiltration of different immune cells, especially CD8^+^ T cells.	Jin et al. [261]
